# Cryptochrome 1 activation inhibits melanogenesis and melanosome transport through negative regulation of cAMP/PKA/CREB signaling pathway

**DOI:** 10.3389/fphar.2023.1081030

**Published:** 2023-02-06

**Authors:** Rongyin Gao, Ximei Zhang, Kun Zou, Duo Meng, Jinpeng Lv

**Affiliations:** ^1^ Department of Pharmacy, Department of Dermatology, The first people’s Hospital of Changzhou, The third Affiliated Hospital of Soochow University, Changzhou, China; ^2^ School of Pharmacy, Changzhou University, Changzhou, China

**Keywords:** cryptochrome 1, KL001, melanogenesis, melanosome transport, cAMP/PKA/CREB pathway

## Abstract

Cutaneous pigmentation was recently shown to be an event regulated by clock proteins. Cryptochrome (CRY) is a key protein composing the feedback loop of circadian clock, however, the function of CRY in melanocytes remains unclear. Here, we found that KL001, a synthetic small molecule modulator of CRY1, inhibited melanin synthesis, as well as reduced melanocyte dendrite elongation and melanosome transport. In addition, the dominant role of CRY1 in KL001-induced anti-melanogenesis was revealed by small interfering RNA transfection. Cellular tyrosinase activity and expression level of melanogenic proteins, including tyrosinase, TRP-1, TRP-2, and transport proteins like Rab27a, Cdc42 and Myosin Va induced by α-MSH were remarkably reversed after KL001 treatment. Mechanistically, CRY1 activation inhibited melanogenesis through CREB-dependent downregulation of MITF and CREB phosphorylation was mediated by classical cAMP/PKA pathway. In addition, the other CRY1 activator, KL044 also suppressed cAMP/PKA/CREB pathway and inhibited melanogenesis. Finally, anti-melanogenic efficacy of KL001 was confirmed by determination of melanin contents in UVB-tanning model of brown guinea pigs, which indicated that targeting CRY1 activity, *via* topical application of small molecule activator, can be utilized therapeutically to manage human pigmentary disorders.

## Introduction

Melanin pigments, naturally produced in melanocytes, is essential in protecting skin from ultraviolet radiation (UVR)-induced damage (Yamaguchi et al*.*, 2006). However, abnormal accumulation of melanin may cause serious dermatological disorders (Ortonne and Passeron, 2005). Strict regulation of melanin deposition is important for controlling hyperpigmentation. Globally, the treatment of hyperpigmentation disorders remains a significant unmet clinical need. Therefore, recognition of novel and crucial mediator is necessary to improve medications for skin pigmentary disorders.

Melanosomes are tissue-specific lysosome-associated organelles in melanocytes, which is responsible for the synthesis and storage of melanin pigments (Koike et al*.*, 2018; Lv et al*.*, 2020a; Lv et al*.*, 2021). Upon UVB exposure, activated melanocytes and keratinocytes locally secrete α-melanocyte-stimulating hormone (α-MSH) (Cui et al*.*, 2007; Ozdeslik et al., 2019). Subsequently, α-MSH binding to Gαs-coupled melanocortin-1 receptor (MC1R) leads to stimulation of adenylyl cyclase (AC) and increase in the concentration of cellular cyclic adenosine monophosphate (cAMP) (Ozdeslik et al*.*, 2019). cAMP-mediated expression of microphthalmia-associated transcription factor (MITF) induces the expression of tyrosinase and tyrosinase-related protein 1 and 2 (TRP-1 and TRP-2) (Lo and Fisher, 2014), thereby driving melanosome maturation and increasing melanin production (Paterson et al., 2015). After maturation, melanosomes are transported from the perinuclear region to the tips of dendrite along actin filaments and microtubules in a coordinated manner by motor proteins (Ohbayashi and Fukuda, 2012).

The circadian clock is an important physiological process for maintaining homeostasis (Duan et al*.*, 2021). It is constructed by a group of conserved clock proteins, including Circadian Locomotor Output Cycles Kaput (CLOCK) and Aryl hydrocarbon receptor nuclear translocator-like protein 1 (BMAL1) representing positive components, while Period (PER) and Cryptochrome (CRY) composing the negative limb of the loop (Panda et al., 2002). An elegant study found that disruption of core clock protein activity increased melanogenesis melanosome transfer in human epidermal melanocytes (Hardman et al., 2015). Although clock proteins such as PER1, BMAL1 and CLOCK all showed the ability to regulate pigmentation process (Hardman et al., 2015; Poletini et al., 2016), the function of CRY in pigmentation remains poorly understood. The CRY family has two members, namely CRY1 and CRY2 (Cashmore et al*.*, 1999). Lately, it has been found that CRY1 protein may exert critical functions in skin cells. In human keratinocytes, CRY1 activity is regulated by blue light and has a positive effect on human hair growth (Buscone et al*.*, 2021). Furthermore, *Cry1* gene expression is severely downregulated while melanogenesis ability is significantly enhanced in B16 melanoma cells in comparison with normal melanocytes (de Assis et al., 2016), suggesting that CRY1 may be involved in melanin deposition. Taken together, these cues encouraged us to examine the function and mechanism of CRY1 in pigmentation.

In the current study, we investigated whether CRY1 activation would affect melanogenesis and melanosome distribution. Firstly, our results showed that KL001, a synthetic small molecule modulator of CRY1, inhibited melanin synthesis, as well as reduced melanocyte dendrite elongation and melanosome transport. Then, the dominant role of CRY1 in KL001-mediated anti-melanogenesis was further confirmed by transfecting small interfering RNA (siRNA). Mechanistically, once CRY1 activated, it reduced cellular cAMP level and inhibited the phosphorylation of PKA and CREB, and subsequently downregulated the expression of MITF and other critical melanogenic and transfer-related proteins, finally inhibited melanin synthesis and distribution.

## Materials and methods

### Materials

KL001 (CAS: 309928-48-1) and KL044 (CAS: 1801856-93-8) were purchased from MedChemExpress (New Jersey, United States of America). Cell lysis buffer (P0013), BCA protein assay kit (P0012) and cAMP assay kit were purchased from Beyotime Biotechnology (Shanghai, China). α-MSH (M118985), ACTH (A118750), Forskolin (F127328) and IBMX (I106812) were obtained from Aladdin (Shanghai, China). Anti-Myosin Va antibody (sc-365986) were purchased from Santa Cruz Biotechnology (Santa Cruz, United States of America). Anti-CRY1 (ab54649), CRY2 (ab93802), tyrosinase (ab180753), TRP-1 (ab235447), TRP-2 (ab74073), Cdc42 (ab187643), Rab27a (ab55667), KIF 5b (ab167429), MITF (ab20663), PKA cat (ab216572), p-PKA cat (ab75991), CREB (ab32515), p-CREB (ab32096), and β-actin (ab8226) antibody were purchased from Abcam (Cambridge, UK). Masson-Fontana melanin staining solution was obtained from SenBeiJia Biological Technology (Nanjing, China).

### Cell culture and siRNA transfection

B16F10 murine melanoma cells from National Collection of Authenticated Cell Culture (Shanghai, China) were maintained in DMEM medium (L110KJ, Basalmedia) containing 10% fetal bovine serum (FBS-S500, NEWZERUM) and penicillin-streptomycin solution (C0222, Beyotime) under 5% CO_2_ at 37°C. B16 cells were transfected with siRNA against mouse CRY1 (sc-44835) and CRY2 (sc-44836) from Santa Cruz Biotechnology as the manufacturer’s instructions.

Normal human epidermal melanocytes (NHEMs) from Sciencell Research Laboratories (CA, United States of America) were maintained in human epidermal melanocyte complete medium (CM-H108, Procell).

### Cell viability test

Cell viability was determined using the MTT assay. Briefly, cells seeded in 96-well plates were treated with KL001 for 48 h. After washing with PBS 3 times, cells were treated with MTT solution (20 μL) for another 4 h. The absorbance was determined at 570 nm.

### Melanin content assay

Intracellular melanin content was utilized as an important indicator of melanin synthesis (Lee et al., 2015; Lv et al., 2019). Briefly, cells seeded in 6-well plates were treated with different concentrations of KL001 in presence or absence of cAMP stimulators such as α-MSH, FSK, ACTH and IBMX. After 48 h, cell pellet was collected and dissolved in 100 μL of 1 mol/L NaOH working solution for 1 h at 80 °C. Melanin content was assessed by a microplate reader at 405 nm.

### Masson–Fontana melanin staining

Samples were fixed and stained as previously reported (Gu et al., 2018). Briefly, slides were incubated in ammoniacal silver solution for 14 h at room temperature. After washing with double distilled water 3 times, slides were incubated in hypo solution for 6 min, followed by counterstain with neutral red stain for another 6 min. Then cell morphology and pigmentation were observed under a Nikon Ti2-U microscope.

### Transmission electron microscopy (TEM)

B16F10 cells were fixed with glutaraldehyde fixing solution (4%) overnight, and post-fixed in 2% osmium tetraoxide in 0.1 M cacodylate for another 1 h at room temperature. Then, B16F10 cells were dehydrated using a graded series of ethanol and embedded in epoxy resin. The regions containing B16F10 cells were cut into ultrathin sections, stained with uranyl acetate and lead citrate, and visualized under TEM (Hitachi, H-7800).

### Scanning electron microscopy (SEM)

B16F10 cells were grown on glass coverslips, fixed in glutaraldehyde fixing solution (4%) for 2 h and post-fixed in osmium tetroxide (1%), dehydrated with ethanol and finally dehydrated overnight with hexamethyldisilazane. Coverslips were gold-coated and visualized under SEM (Hitachi, Regulus-8100).

### Cellular and cell-free tyrosinase activity assay

Cellular tyrosinase activity was measured following a previously described method (Lv et al*.*, 2020b; Lv et al*.*, 2021). Briefly, B16F10 cells were treated with different concentrations of KL001 (0, 5, 10, 20 μM) for 48 h. Cells were washed with cold PBS and lysed in lysis buffer. Then lysates were centrifuged to acquire the supernatant to determine tyrosinase activity and quantify protein levels. 100 μL PBS (0.1 M, pH 6.5) contenting 10 μg proteins mixed with 100 μL 0.1% L-DOPA. Optical absorbance was monitored at 475 nm using a microplate reader after incubation at 37°C for 1 h.

Cell-free tyrosinase activity was performed as the previous description (Lv et al*.*, 2020b; Lv et al*.*, 2021). Briefly, 100 μL of PBS (0.1 M, pH 6.5) containing different concentrations of KL001 (0, 5, 10, 20 μM) was mixed with mushroom tyrosinase (10 unit) and 50 μL of 0.03% L-tyrosine. Optical absorbance was monitored at 475 nm using a microplate reader after incubation at 37°C for 10 min.

### Enzyme-linked immunosorbent assay

B16F10 cells were pretreated with KL001 (or KL044) for 2 h and stimulated with FSK for 10 min in the presence of KL001 (or KL044). The cells were lysed with Triton X-100 (1%) at 4 °C for 1 h, and the lysates were clarified by centrifugation at 12,000 rpm for 20 min at 4 °C. The supernatant was used to assess the level of cAMP using cAMP ELISA Kit (E-EL-0056c, Elabscience Biotechnology), following the manufacturer’s instructions.

### Western blot

Western blot activity was performed following a previously described method (Lv et al., 2021). Briefly, protein samples were separated using SDS polyacrylamide gel electrophoresis, and then transferred using an electrophoretic transfer technique onto nitrocellulose filter membranes. After blocking with 3% BSA in TBST solution for 1.5 h at room temperature, cut horizontally to incubate with suitable primary antibodies for 12 h at 4°C. After washing with TBST 4 times, the membranes were incubated with peroxidase-conjugated secondary antibodies for 1 h at room temperature, and visualized using enhanced chemiluminescence. The membranes were subsequently stripped and reprobed with specific antibodies. Data reported here represent at least three independent replicates.

### Melanin content determination in UVB-induced hyperpigmentation model in brown Guinea pig

The animal experiment scheme in this work was approved by the animal care and use committee of Changzhou University (CZDX-2021009). Eight brown guinea pigs (∼300 g, 6 weeks) were obtained from the Institute of Laboratory Animal Science (Beijing, China). The animals were housed individually in rooms with constant temperature, humidity and a 12-h light and dark cycle. After adaptation, UVB-induced hyperpigmentation model was performed on the back of brown guinea pigs as previous description (Lee et al*.*, 2013; Lv et al*.*, 2020c; Lv et al*.*, 2021). The control group was applied with vehicle (PEG400/EtOH = 7:3) and the treatment group was applied with KL001 (1%) on the hyperpigmented areas (20 μL per circle) twice a day for 4 weeks. The L-value was detected by spectrophotometer (YS3010, 3nh, Shenzhen, China) to calculate the ΔL-value, which was utilized to evaluate the degree of pigmentation. The calculation formula is shown below: ΔL = L (daily measured)-L (Day 0) (Lee et al., 2013; Lv et al*.*, 2020b).

### Statistical analysis

Values were expressed as mean ± SEM, and statistical comparisons were analyzed by GraphPad Prism using one-way ANOVA, followed by Turkey’s *post hoc* test for multiple comparison tests. When *p* < 0.05, the difference was statistically significant.

## Results

### Stimulation of CRY1 by KL001 treatment inhibited melanogenesis in B16F10 cells

**FIGURE 1:**
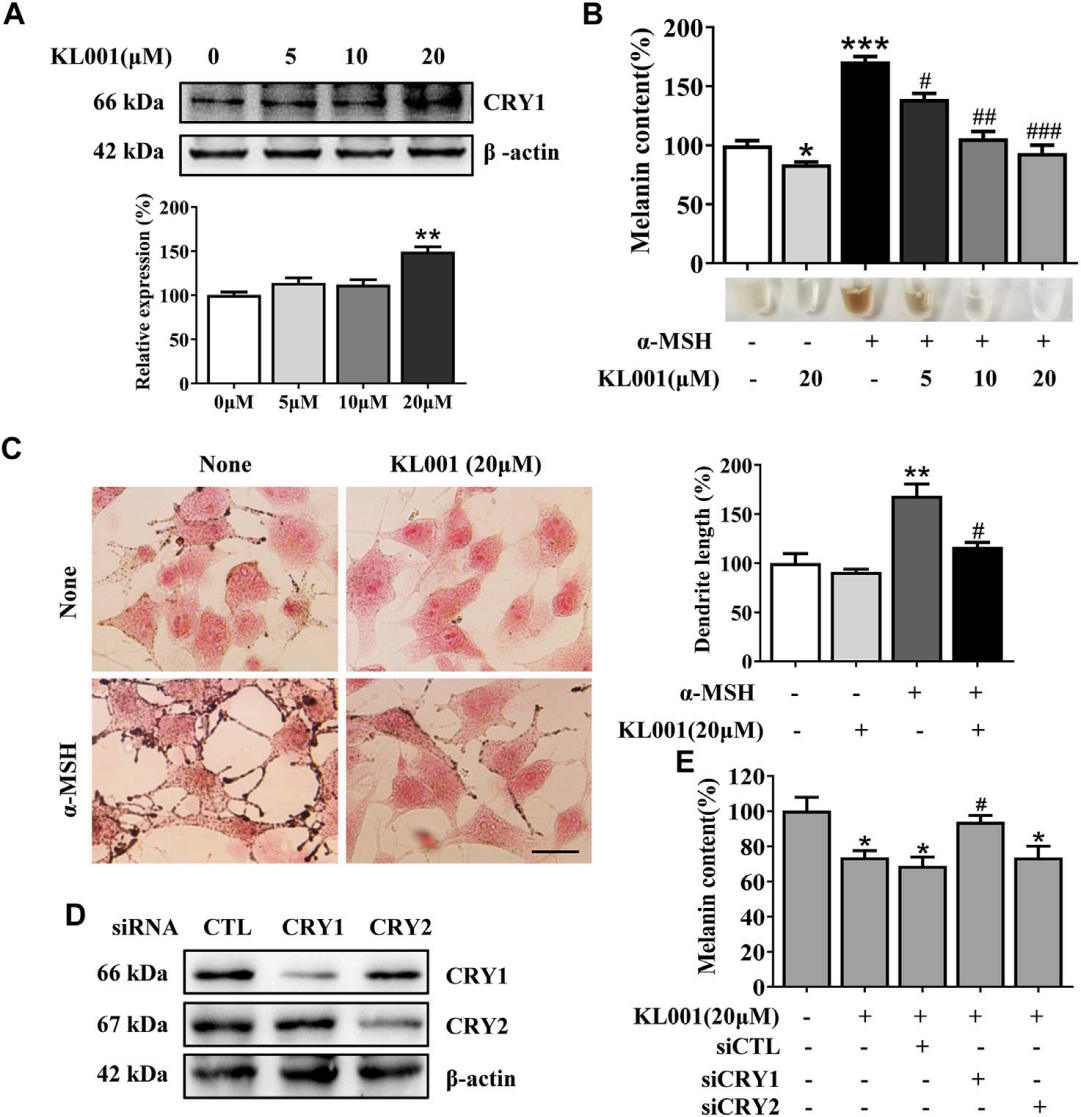
KL001 inhibited melanogenesis in B16F10 cells. **(A)** Western blot analysis to detect CRY1 protein levels were performed after treatment with KL001 for 48 h. **(B)** Melanin contents and **(C)** Fontana-Masson staining photograph are presented after treatment with KL001 in presence or absence of α-MSH for 48 h. Bar = 20 μm. Total length of dendrites per cell was measured on the pictures using ruler. **(D, E)** B16F10 cells were transfected with siRNA oligonucleotides using transfection reagent. WB analysis was performed after 48 h, and melanin contents were measured after 48 h in the presence of KL001. Data are expressed as the mean ± SD (*n* = 3). ^*^ *p* < 0.05, ^**^ *p* < 0.01, ^***^ *p* < 0.001 versus non-treated cells. ^#^ *p* < 0.05, ^##^ *p* < 0.01, ^###^ *p* < 0.001 versus treated cells with indicated reagents.

KL001 is identified as a small molecule activator of CRY (Hirota et al., 2012). To investigate whether CRY1 protein is activated after KL001 treatment in B16F10 cells, CRY1 protein level was determined by western blotting. As shown in Figure 1A, 20 μM KL001 markedly increase CRY1 protein expression in B16F10 cells. In addition, KL001 exerted no cytotoxicity up to 20 μM for 48 h (Supplementary Figure S1). These results revealed that CRY1 was regulated by KL001 treatment in B16F10 cells.

Next, the effect of KL001 on melanogenesis was determined by a melanin content assay and Masson-Fontana ammoniacal silver staining. As shown in Figure 1B, KL001 suppressed the basal melanogenesis and reversed α-MSH (100 nM)-induced melanin increase. Consistently, Fontana-Masson staining revealed that KL001 significantly reduced melanin concentration in dendrites and perinuclear region, as well as the increased dendrite number and length in presence or absence of α-MSH (Figure 1C).

Finally, the crucial role of CRY1 in KL001-induced anti-melanogenesis was demonstrated by effectively transfecting siRNA. CRY1 siRNA (or CRY2 siRNA) significantly reduced CRY1 (or CRY2) mRNA levels over 80% in the B16F10 cells (Figure S2). Silencing CRY2 did not reverse the effects of KL001, whereas KL001 treatment failed to inhibit melanogenesis in the absence of CRY1 (Figures 1D, E), reflecting that CRY1 activation by KL001 treatment suppressed melanogenesis in B16F10 cells.

### KL001 inhibited the maturation of melanosomes in B16F10 cells

**FIGURE 2:**
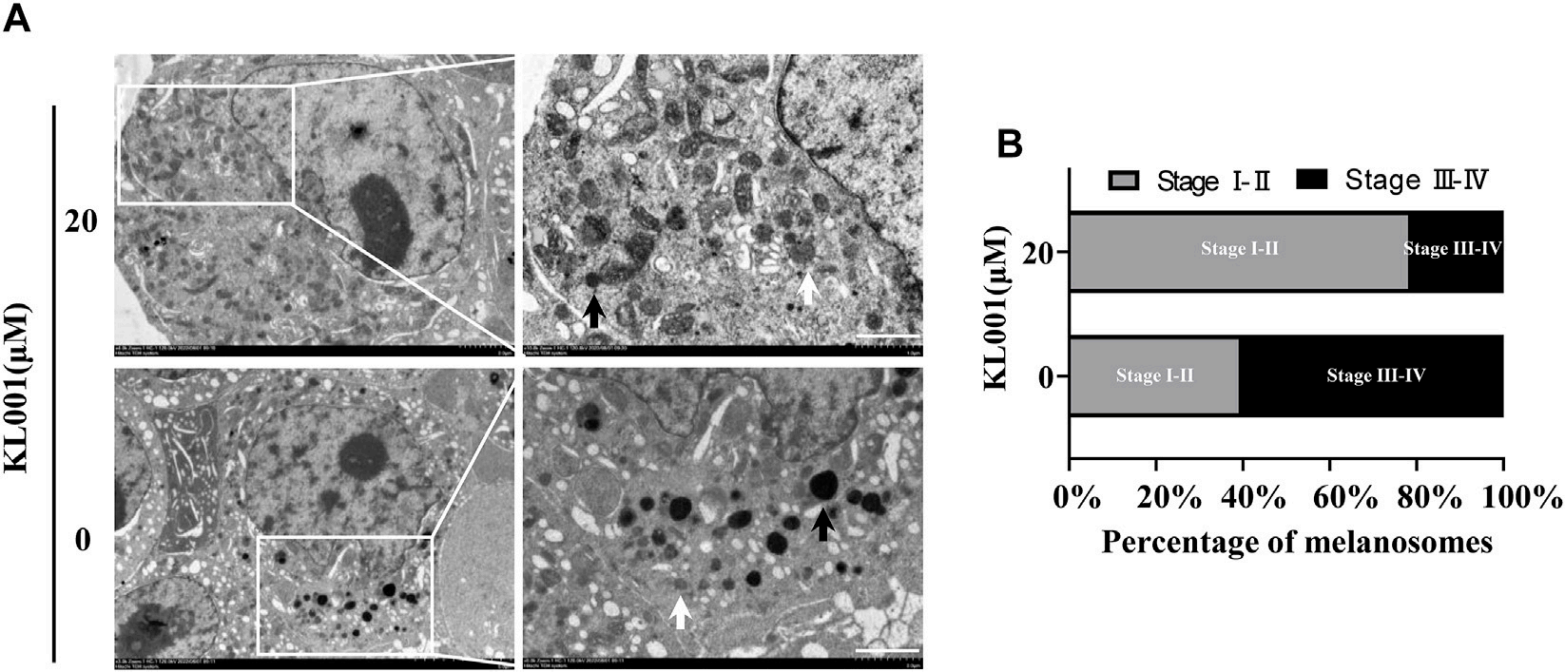
KL001 inhibited melanosome maturation. **(A)** B16F10 cells under TEM after 48 h in the presence of KL001. The white arrow and the black arrow indicated stage I-II and stage III-IV melanosomes respectively. Bar = 1 μm. **(B)** Stage I-II and stage III-IV melanosome numbers presented as percentage of all. Data are expressed as the mean ± SD (*n* = 3).

Melanogenesis is closely related to the maturation of melanosomes (Raposo and Marks, 2007). To explore the effect of KL001 on melanosome maturation, melanosome stage was observed under TEM. As shown in Figure 2, stage III-IV melanosomes were significantly reduced in B16F10 cells after KL001 treatment, suggesting that KL001 inhibited the maturation of melanosomes.

### KL001 inhibited the cellular activity of tyrosinase and decreased melanogenic proteins expression

**FIGURE 3:**
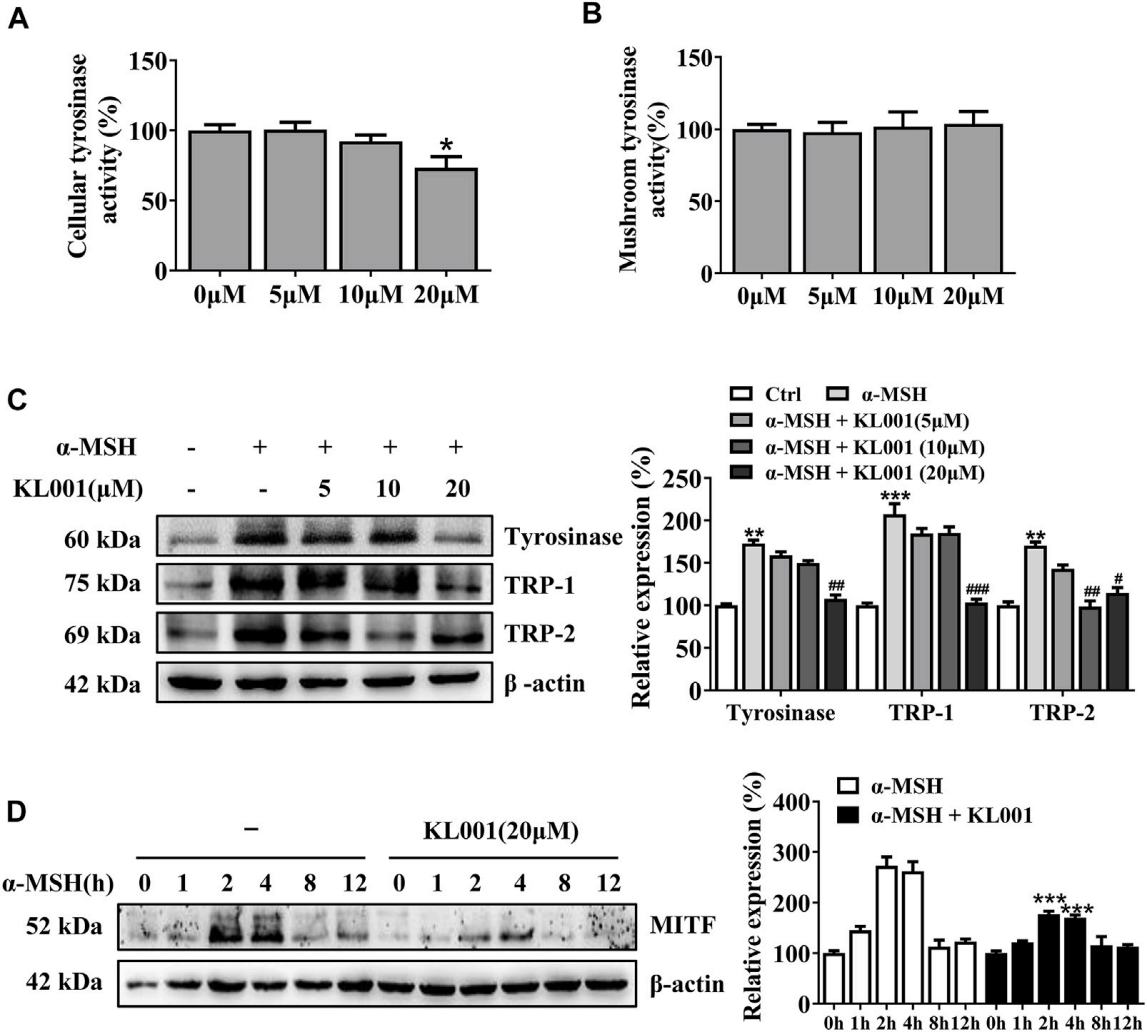
KL001 inhibited tyrosinase activity and expression of melanogenic proteins in B16F10 cells. **(A)** Cellular tyrosinase activity and **(B)** Mushroom tyrosinase activity were determined as described in methods. **(C)** Protein expression levels of tyrosinase, TRP-1 and TRP-2 were measured by western blotting after treatment with KL001 for 48 h. **(D)** MITF protein expression was analyzed after treatment with α-MSH in the presence or absence of KL001 for different times. Data are expressed as the mean ± SD (*n* = 3). ^*^ *p* < 0.05, ^**^ *p* < 0.01, ^***^ *p* < 0.001 versus non-treated cells. ^#^ *p* < 0.05, ^##^ *p* < 0.01, ^###^ *p* < 0.001 versus α-MSH-treated cells.

Tyrosinase activity is necessary in melanogenesis (Lv et al., 2020a; Lv et al., 2021). L-DOPA oxidation and mushroom tyrosinase activity assay were used to determine the effect of KL001 on cellular and cell-free tyrosinase activity, respectively. Consistent with the anti-melanogenic effect, 20 μM KL001 significantly inhibited cellular activity of tyrosinase (Figure 3A). However, KL001 showed no effect on the enzymatic activities of mushroom tyrosinase (Figure 3B).

To determine whether the efficacy of KL001 was relative to tyrosinase, TRP-1 and TRP-2 protein, three key melanogenic enzymes, the protein expression levels were examined (Lv et al., 2019). As shown in Figure 3C, with α-MSH stimulation, KL001 significantly reduced the expression of tyrosinase, TRP-1 and TRP-2, indicating that the whitening effects of KL001 is mediated by the suppression of cellular tyrosinase activity and associated key melanogenic protein expression.

MITF is an essential transcription factor that induces gene expression of tyrosinase, TRP-1 and TRP-2 (Lv et al., 2020b), therefore the MITF dynamics following KL001 treatment were monitored. As shown in Figure 3D, following α-MSH stimulation, expression of MITF protein peaked at 2 h, and KL001 treatment significantly decreased it, which indicated that KL001 decreased α-MSH-induced tyrosinase, TRP-1 and TRP-2 expression through inhibiting MITF expression.

### KL001 inhibited melanosome distribution by reducing Myosin Va, Rab27a and Cdc42 expression

**FIGURE 4:**
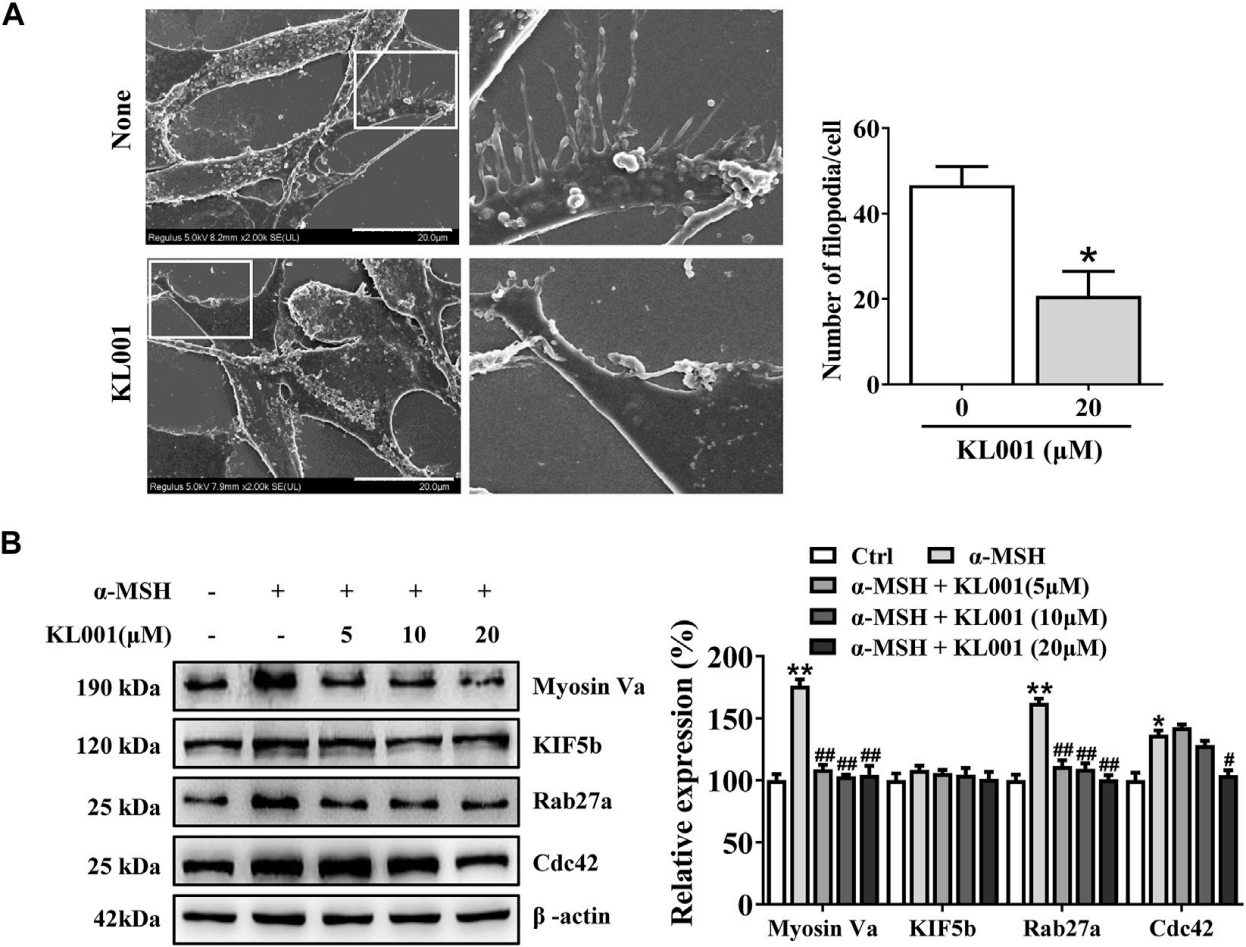
KL001 inhibited filopodia formation and expression of melanosome transport proteins in B16F10 cells. **(A)** SEM quantification of filopodia numbers in B16F10 cells. 10 cells/condition were assessed in each of 3 independent experiments. Bar = 20 μm. **(B)** The expression levels of related melanin transport proteins were analyzed. Data are expressed as the mean ± SD (*n* = 3). *p < 0.05, **p < 0.01versus non-treated cells. #*p* < 0.05, ##*p* < 0.01 versus α-MSH-treated cells.

In melanocytes, melanin is produced in the cell body, transported to the dendrites, and finally to neighboring keratinocytes to complete distribution. As shown by Fontana-Masson staining, KL001 significantly decreased dendrite formation and the amount of melanin pigments in dendrites (Figure 1C). Furthermore, SEM results revealed that KL001 remarkedly inhibited melanocyte filopodia formation (Figure 4A).

To further elucidate the underlying mechanism, several key factors involved in melanosome distribution were determined. Firstly, Kinesin superfamily protein 5b (KIF5b) is contributed to the outward transport of melanosome along microtubules (Noguchi et al., 2014). While melanosomes transfer from microtubules to actin filaments, melanosomes are transported by the tripartite complex consisting of Rab27a-Mlph-myosin Va and then anchored to the plasma membrane by the Rab27a- Slp2-a complex (Ohbayashi and Fukuda, 2012; Ishida et al*.*, 2014; Fukuda, 2021). In addition, Cdc42 contributes to dendrite extension and filopodia formation (Lv et al., 2020b). As shown in Figure 4B, Myosin Va, Rab27a and Cdc42 protein expression were significantly decreased after KL001 treatment, but not KIF5b, indicating that KL001 inhibited melanosome distribution by reducing Myosin Va, Rab27a and Cdc42 expression.

### CAMP/PKA/CREB pathway contributes to KL001-induced anti-melanogenesis

Recently, several studies have reported that the biological effects of CRY1 depend on the negative regulation of cAMP/PKA signaling pathway (Zhang et al., 2010; Narasimamurthy et al., 2012; Qin and Deng, 2015; Yamanaka et al., 2018; Huang et al*.*, 2020). Therefore, it is worth investigating whether KL001 affects melanogenesis induced by other cAMP activators. Briefly, adrenocorticotropic hormone (ACTH) is an endogenous agonist of MC1R (Pillaiyar et al*.*, 2017b), forskolin (FSK) is a direct stimulator of adenyl cyclase to produce cAMP, and 3-isobutyl-l-methylxanthine (IBMX) is a cAMP phosphodiesterase inhibitor that prevent the degradation of cAMP (Shin et al*.*, 2015). As expected, KL001 treatment also inhibited ACTH (100 nM)-, FSK (10 μM)- and IBMX (100 μM)-induced melanogenesis in B16F10 cells (Figure S3).

**FIGURE 5:**
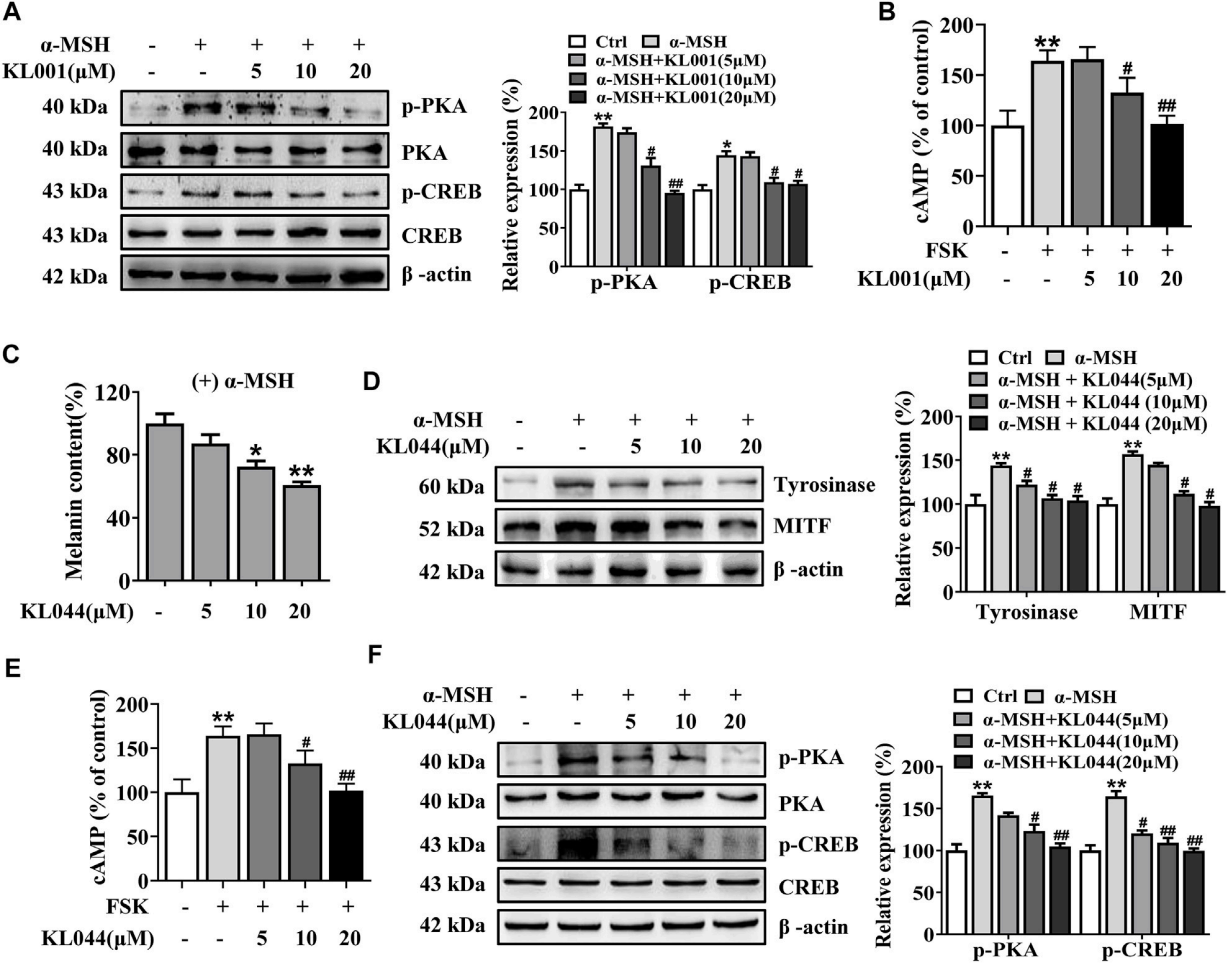
KL001 and KL044 suppressed cAMP/PKA/CREB signaling pathway in B16F10 cells. **(A)** Phosphorylation of PKA and CREB, **(B)** Cellular cAMP levels were detected. B16F10 cells were pretreated with KL001 for 2 h and stimulated with α-MSH for 20 min or FSK for 10 min in the presence of KL001. **(C–F)** Melanogenesis efficacy and related signaling pathway of KL044 were detected. Data are expressed as the mean ± SD (*n* = 3). ^*^ *p* < 0.05, ^**^ *p* < 0.01 versus non-treated cells. ^#^ *p* < 0.05, ^##^ *p* < 0.01 versus α-MSH- or FSK-treated cells.

Further, whether the cAMP/PKA pathway is activated after KL001 treatment in melanocytes is another key question that needs to be investigated. As shown in Figure 5A and Supplementary Figure S4, KL001 induced a decrease in the phosphorylation of CREB and PKA in presence of α-MSH. These results indicated that KL001 treatment can inhibit CREB-dependent MITF induction, which can influence melanogenic enzymes expression. In addition, the increased cellular cAMP level induced by FSK was also reduced after KL001 treatment (Figure 5B). Based on the above results, it can be speculated that KL001 treatment regulated the intracellular cAMP levels to regulate the subsequent downstream signaling pathway.

In addition, we investigated whether the whitening effects were shared by the other CRY activator. KL044 is a KL001 analogue with higher potency (Lee et al., 2015). In melanin content assay, KL044 also strongly suppressed melanin synthesis induced by α-MSH in a dose-dependent manner (Figure 5C). Expression level of tyrosinase and MITF was markedly decreased after KL044 treatment (Figure 5D). Mechanistically, KL044 strongly inhibited cellular cAMP production and subsequent activation of PKA/CREB signaling pathway (Figures 5E, F). These results confirmed that the activation of CRY inhibited melanogenesis through negative regulation of cAMP/PKA/CREB pathway.

### KL001 suppressed melanogenesis in normal human epidermal melanocytes

**FIGURE 6:**
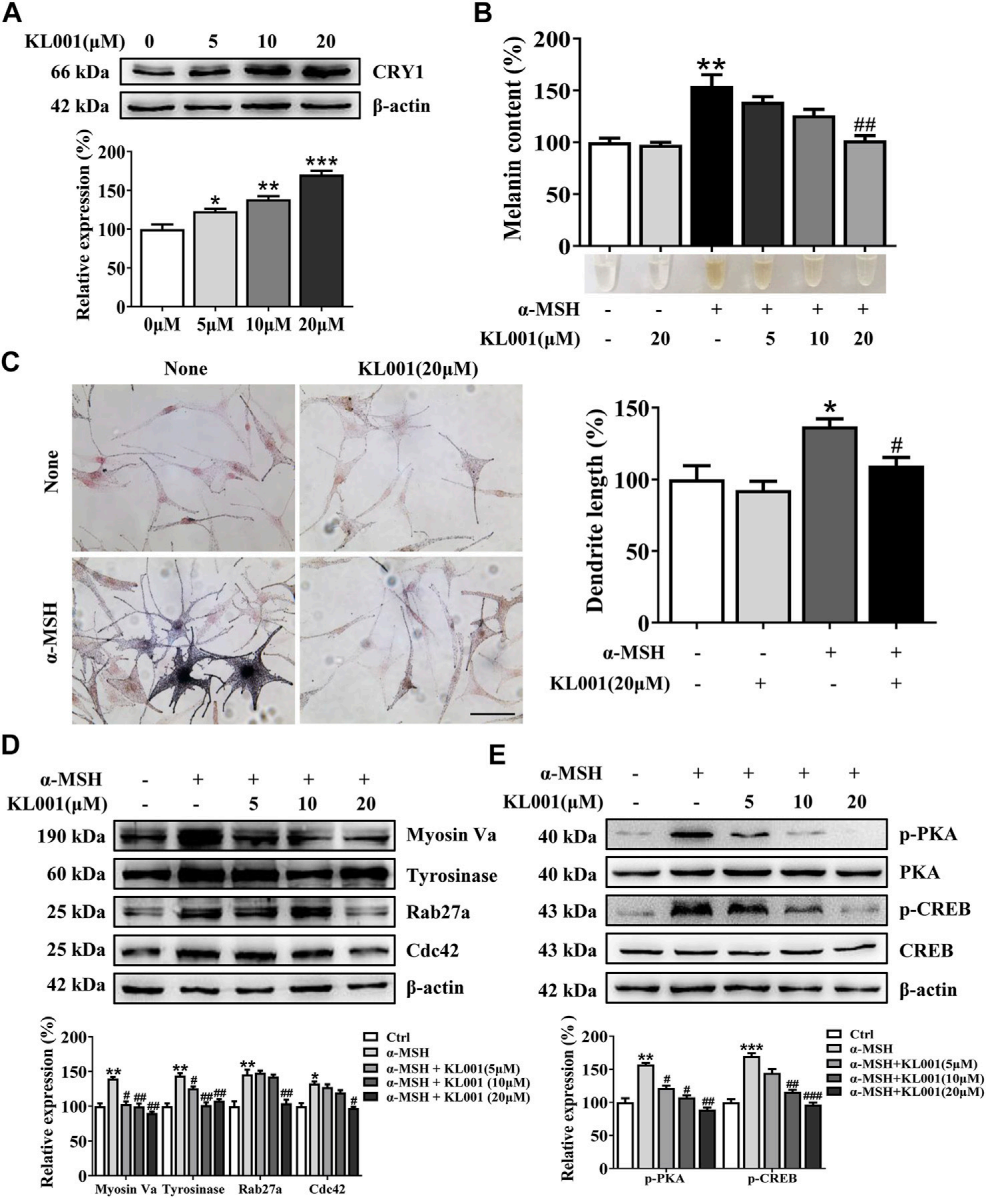
KL001 inhibited melanogenesis in human melanocytes. **(A)** Human melanocytes were treated with KL001 for 48 h and CRY1 expression was measured by western blot analysis. **(B)** Melanin contents were measured as described in methods. **(C)** Human melanocytes were stained with Fontana-Masson staining. Bar = 20 μm. Total length of dendrites per cell was measured on the pictures using ruler. **(D)** Expression of tyrosinase, Myosin Va, Cdc42 and Rab27a were measured using western blotting as described in methods. **(E)** Phosphorylation level of PKA and CREB were detected. Human melanocytes were pretreated with KL001 for 2 h and stimulated with α-MSH for 20 min in the presence of KL001. Data are expressed as the mean ± SD (*n* = 3). ^*^ *p* < 0.05, ^**^ *p* < 0.01, ^***^ *p* < 0.001 versus non-treated cells. ^#^ *p* < 0.05, ^##^ *p* < 0.01, ^###^ *p* < 0.0001 versus α-MSH -treated cells.

The inhibitory efficacy of KL001 on melanogenesis was investigated in primary normal human epidermal melanocytes (NHEM). As shown in Figure 6A, KL001 treatment increased the expression of CRY1 protein in NHEM. Consistent with data from B16F10 cells, KL001 treatment also reduced α-MSH-induced melanin in dendrites and perinuclear region (Figures 6B, C). Western blotting results indicated that KL001 decreased the expression of tyrosinase, Cdc42, Myosin Va and Rab27a in NHEM (Figure 6D). Mechanistically, KL001 strongly inhibited the PKA/CREB signaling pathway (Figure 6E), confirming that the anti-melanogenic efficacy of KL001 in NHEM was mediated by PKA/CREB signaling pathway.

### KL001 suppressed UVB-induced hyperpigmentation in brown Guinea pig

**FIGURE 7:**
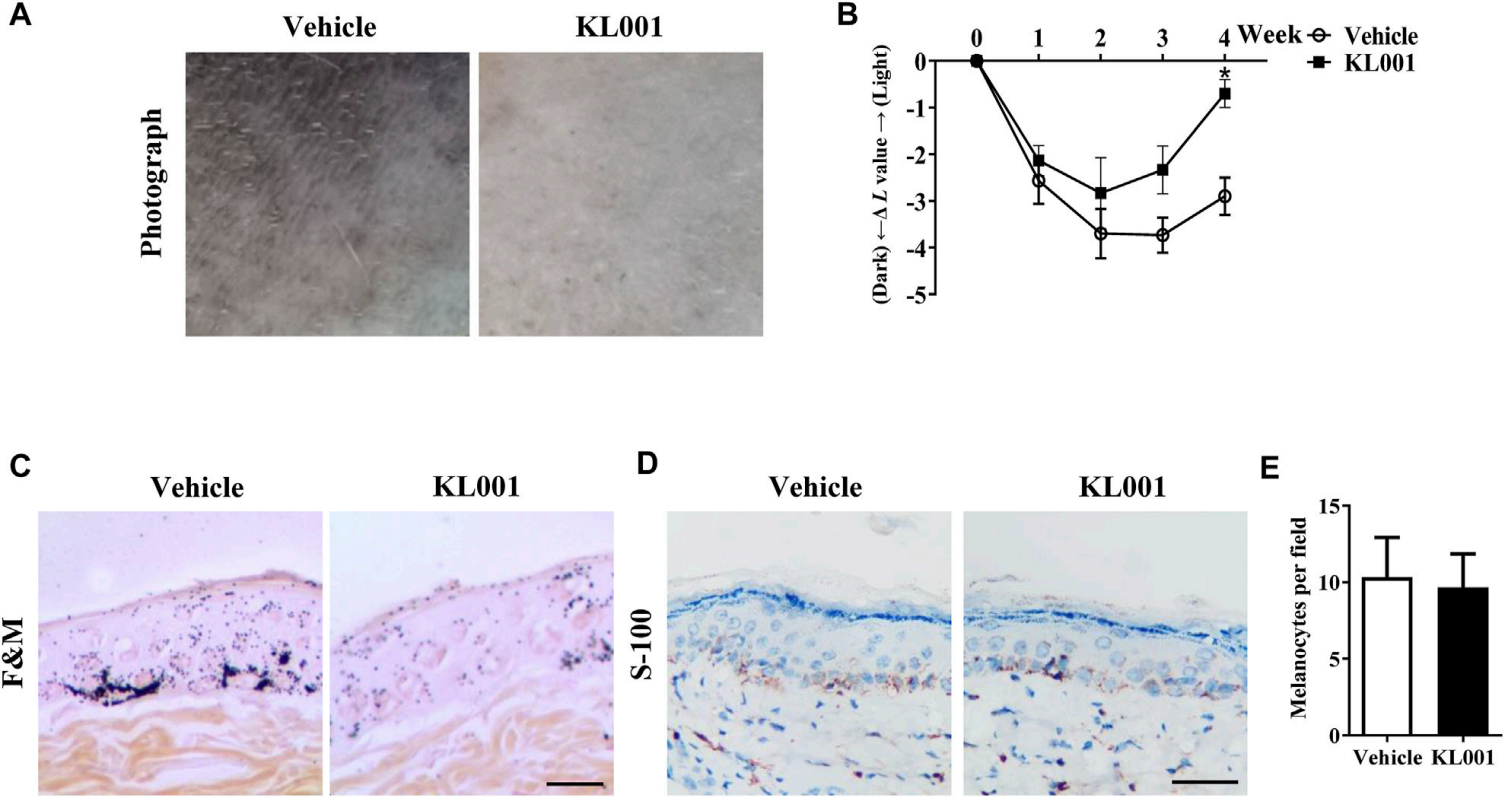
KL001 reduced UVB-induced hyperpigmentation in brown guinea pig. **(A)** Representative photographs of dorsal skin. **(B)** The degree of depigmentation was determined by a chromameter follows: ΔL = L (at each day measured)—L (at day 0). An increase in the ΔL value indicates a decrease in hyperpigmentation induced by UV. **(C)** Fontana-Masson staining of skin biopsies. **(D, E)** Immunohistochemical staining of skin biopsies for the detection of S-100 as a melanocyte marker protein. Bar = 50 μm ^*^ *p* < 0.05 versus non-treated cells.

The anti-melanogenic efficacy of KL001 was also investigated *in vivo* in UVB-induced hyperpigmentation model in brown guinea pigs. Firstly, as shown by representative photographs, skin pigmentation was significantly suppressed after KL001 (1%) treatment (Figure 7A). To further illustrate the pigmentation degree, L value is calculated by Spectrophotometer and utilized as an index of brightness. As shown in Figure 7B, the ΔL value in KL001 group was significantly higher after 4 weeks, reflecting that KL001 reversed UVB-induced hyperpigmentation. Consistently, hyperpigmentation in the epidermal basal layer was visibly reduced after treatment with KL001 as shown by Fontana-Masson staining (Figure 7C). In addition, immunohistochemical staining of melanocyte marker protein S100 showed that the amounts of melanocyte were not affected by KL001 treatment (Figures 7D, E). Taking together, these observations indicated that KL001 has whitening efficacy on UVB-induced hyperpigmentation *in vivo*.

## Discussion

The presence of clock proteins in skin cells was first reported in 2000 (Zanello et al., 2000), and skin pigmentation was recently shown to be a clock regulated event (Hardman et al., 2015). Clock proteins such as PER1, BMAL1 and CLOCK all showed the ability to regulate pigment metabolism (McClung et al., 2005; Hardman et al., 2015), however, the function of CRY in melanocytes escaped the notice of previous investigators. In this work, we found that activation of CRY, especially CRY1, inhibited α-MSH-induced expression of tyrosinase and cellular tyrosinase activity but not directly affected the catalytic activity of cell-free tyrosinase, ultimately inhibiting melanogenesis (Figures 1, 3). Interestingly, results showed that CRY1 activation suppressed basal melanin synthesis in B16F10 melanocytes (Figure 1), but not in NHEM (Figure 6). This difference may be due to the different expression level of *Cry1* between the malignant and normal melanocytes (de Assis et al., 2016). Most importantly, topical application of KL001 exerted a significant whitening efficacy on UVB-induced hyperpigmentation in brown guinea pigs *in vivo* (Figure 7), indicating that CRY1 might be a crucial intrinsic factor in regulating pigmentation.

Melanosome transport is as important as melanogenesis. As shown by Fontana-Masson staining, KL001 decreased the number of dendrites and melanin pigments in dendrites (Figure 1C). Furthermore, SEM results indicated that KL001 remarkedly inhibited melanocyte filopodia formation (Figure 4A). KIF5b has previously been implicated in the transport of outward melanosomes along microtubules within melanocytes. As melanosomes migrate from microtubules to the actin filaments, their movement were regulated by Myosin Va, and were anchored to the periphery of cell through Rab27a (Chang et al., 2012; Koike et al., 2018). Cdc42 is essential for dendrite extension and filopodia formation, which is required for melanosome transfer. (Lv et al., 2020b). Western blotting results revealed that KL001 significantly decreased Rab27a, Myosin Va and Cdc42 expression. However, KIF5b expression was almost unchanged (Figures 4B, 6D). On the one hand, these results suggested that KL001 inhibited melanosome movement along actin filaments and dendrite extension by decreasing Rab27a, Myosin Va and Cdc42 expression. On the other hand, whether KL001 affects melanosome movement along microtubules needs further study.

MITF is an essential transcription factor that induces gene expression of tyrosinase and tyrosinase-related proteins expression, which is inducible in response to cAMP through the CREB pathway (Lee et al., 2011). Our present work found that KL001 inhibited α-MSH-induced melanogenesis through decreasing MITF expression (Figure 3D). In addition, KL001 suppressed α-MSH-induced CREB activating pathway, directing inhibiting the phosphorylation of CREB, as well as the upstream protein PKA (Figure 5A). Several studies have reported that CRY1 had an inhibitory effect on cAMP/PKA pathway, however, the exact target is still controversial. Two studies reported that the inhibitory target of CRY1 should be the upstream of AC, and one of them directly pointed out CRY1 interacted with Gsα subunit (Zhang et al., 2010; Huang et al., 2020). While another two studies found that CRY1 directly bound to AC and inhibited its activity (Narasimamurthy et al., 2012; Yamanaka et al., 2018). In this work, results firstly showed that KL001 not only alleviated the melanogenic effects of α-MSH and ACTH, but also FSK and IBMX (Figure S3), suggesting that the target of CRY1 protein in the cAMP/PKA pathway should not be the upstream of AC. Secondly, the increased cellular cAMP level induced by FSK was significantly decreased after KL001 treatment (Figure 5B), which indicated that CRY activation regulated cellular cAMP level to control the subsequent signaling pathway. However, the exact target of CRY1 in pigmentation requires further studies.

A number of anti-melanogenic small molecule compounds have been identified *in vitro* studies, however, few agents showed favorable efficacy *in vivo* studies (Pillaiyar et al*.*, 2017a). Therefore, the whitening efficacy of KL001 *in vivo* in the skin of guinea pigs was investigated. As shown in Figure 7, topical application of KL001 showed obvious whitening effects in UVB-induced hyperpigmentation model in guinea pig. Specifically, our results showed that KL001 reversed UVB-induced melanogenesis in active melanocytes, instead of decreasing melanocyte numbers.

**FIGURE 8:**
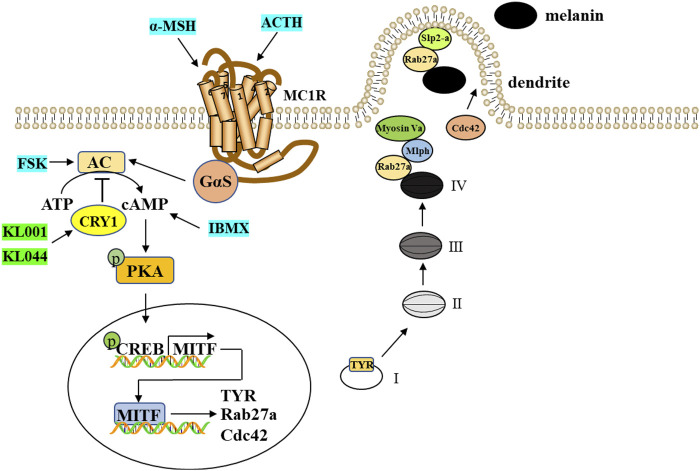
A proposed scheme shows that CRY1 activation inhibits melanogenesis and melanosome transport through negative regulation of cAMP/PKA/CREB signaling pathway. cAMP-mediated expression of MITF induces the expression of TYR, Cdc42, and Rab27a, thereby driving melanosome maturation and transport. TYR is a critical enzyme for the melanogenesis and Cdc42 contributes to dendrite extension and filopodia formation. Rab27a interacts with two Rab27a effectors, Mlph and Slp2-a, and thereby regulates actin-dependent melanosome transport and melanosome anchoring to the plasma membrane, respectively.

In conclusion, our present work reported for the first time that CRY1 activation inhibited melanin deposition by down-regulating key melanogenic proteins *via* negative regulation of cAMP/PKA/CREB signaling pathway (Figure 8). Our findings provided a line of evidence supporting that topical application of small molecule modulators targeting CRY activity can be used therapeutically to manage pigmentary disorders.

## Data Availability

The original contributions presented in the study are included in the article/supplementary material, further inquiries can be directed to the corresponding author.
