# Supplementary material for: Cryptochrome 1 activation inhibits melanogenesis and melanosome transport through negative regulation of cAMP/PKA/CREB signaling pathway
Source: Front Pharmacol. 2023 Feb 6;14:1081030. doi: 10.3389/fphar.2023.1081030 (PMC9939694; doi:10.3389/fphar.2023.1081030)
Supplement: Supplementary file 1 [file Presentation1.PPTX]

## Slide 1
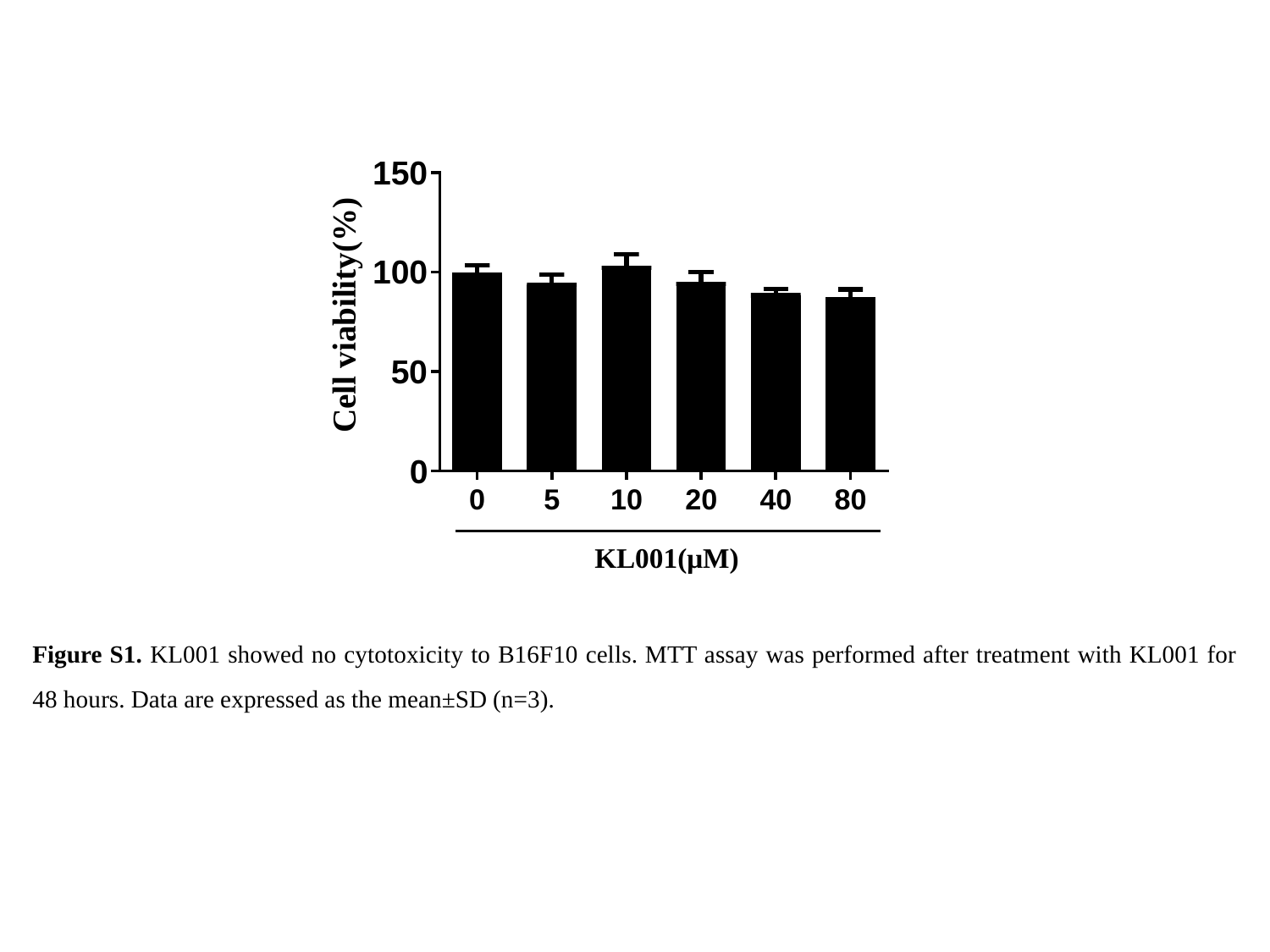

KL001(μM)
Figure S1. KL001 showed no cytotoxicity to B16F10 cells. MTT assay was performed after treatment with KL001 for 48 hours. Data are expressed as the mean±SD (n=3).

## Slide 2
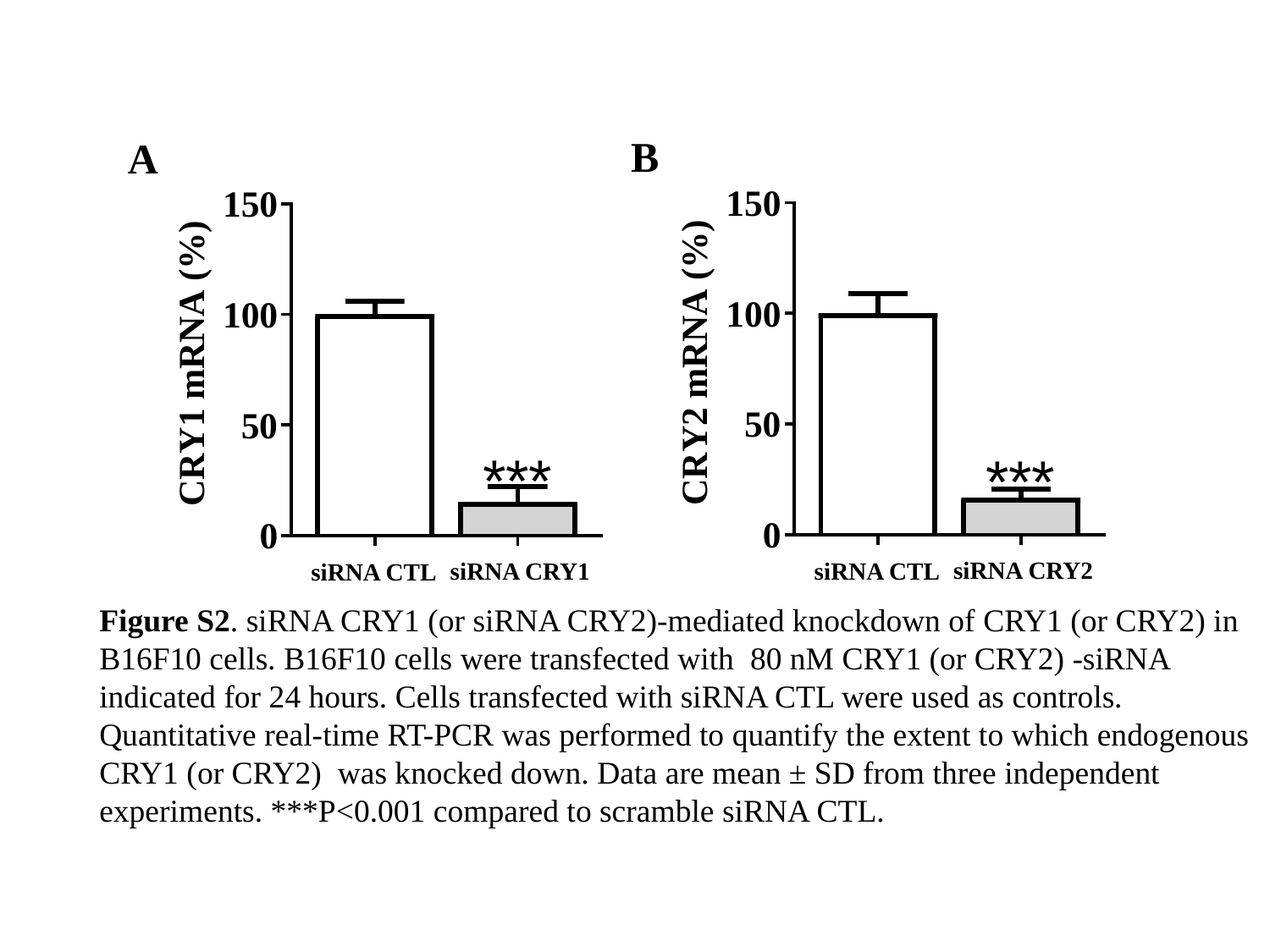

B
A
 siRNA CRY2
 siRNA CRY1
 siRNA CTL
 siRNA CTL
Figure S2. siRNA CRY1 (or siRNA CRY2)-mediated knockdown of CRY1 (or CRY2) in
B16F10 cells. B16F10 cells were transfected with 80 nM CRY1 (or CRY2) -siRNA indicated for 24 hours. Cells transfected with siRNA CTL were used as controls. Quantitative real-time RT-PCR was performed to quantify the extent to which endogenous CRY1 (or CRY2) was knocked down. Data are mean ± SD from three independent experiments. ***P<0.001 compared to scramble siRNA CTL.

## Slide 3
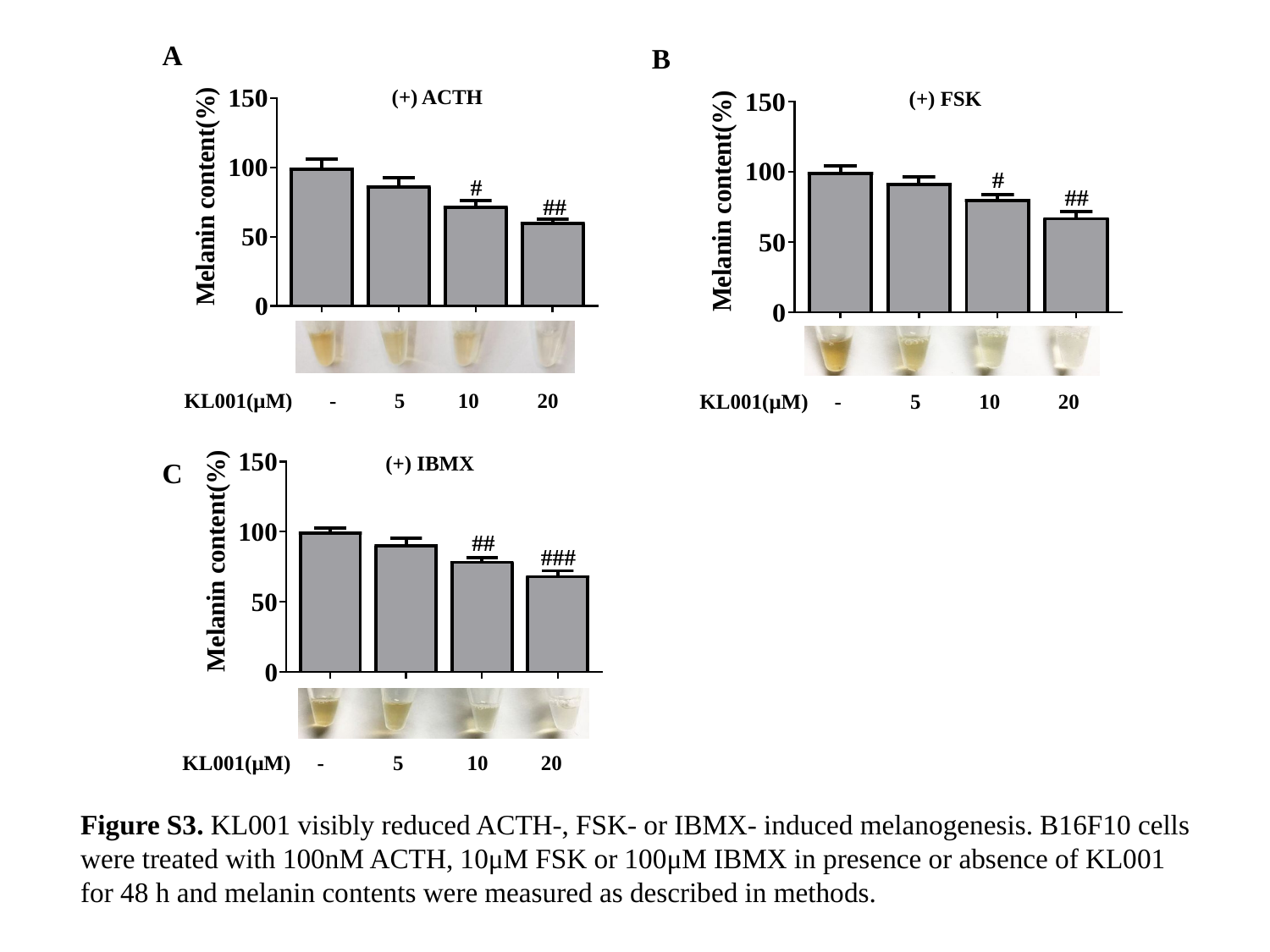

A
B
(+) FSK
(+) ACTH
 KL001(μM) - 5 10 20
 KL001(μM) - 5 10 20
(+) IBMX
C
 KL001(μM) - 5 10 20
Figure S3. KL001 visibly reduced ACTH-, FSK- or IBMX- induced melanogenesis. B16F10 cells were treated with 100nM ACTH, 10μM FSK or 100μM IBMX in presence or absence of KL001 for 48 h and melanin contents were measured as described in methods.

## Slide 4
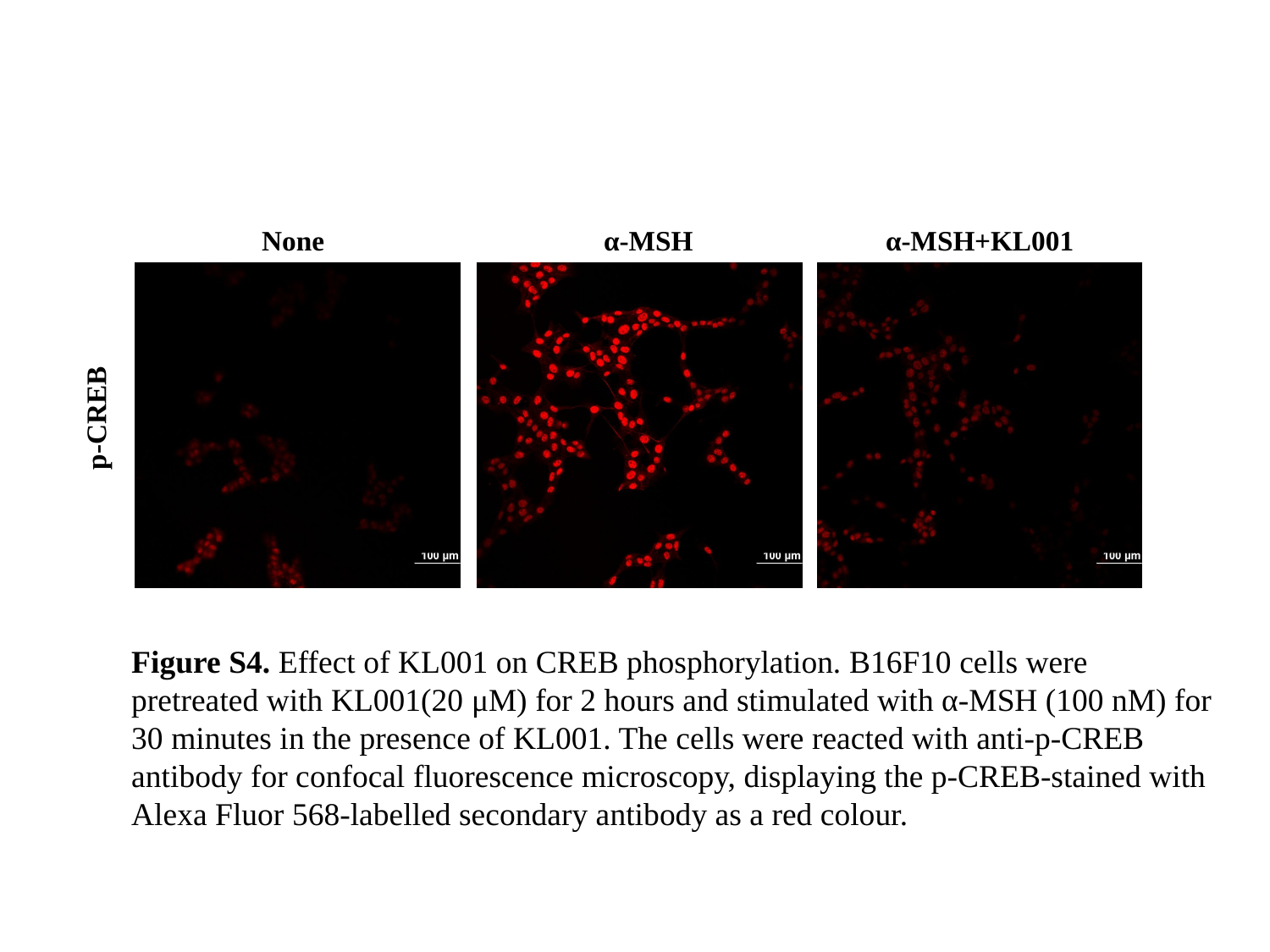

None
α-MSH
α-MSH+KL001
p-CREB
Figure S4. Effect of KL001 on CREB phosphorylation. B16F10 cells were pretreated with KL001(20 μM) for 2 hours and stimulated with α-MSH (100 nM) for 30 minutes in the presence of KL001. The cells were reacted with anti-p-CREB antibody for confocal fluorescence microscopy, displaying the p-CREB-stained with Alexa Fluor 568-labelled secondary antibody as a red colour.
